# Comment on “The Gut Microbiome Profile in Obesity: A Systematic Review”

**DOI:** 10.1155/2018/6015278

**Published:** 2018-08-23

**Authors:** Nuha Alwardat, Laura Di Renzo, Antonino De Lorenzo

**Affiliations:** ^1^PhD School of Applied Medical-Surgical Sciences Faculty of Medicine and Surgery, University of Rome “Tor Vergata”, Rome, Italy; ^2^Division of Clinical Nutrition and Nutrigenomics, Department of Biomedicine and Prevention, University of Rome “Tor Vergata”, Rome, Italy

We carefully read the article by Castaner et al. [1], which was published recently. The topic of the review is interesting, and much work and effort were put into this study to evaluate the association between intestinal microbiota and obesity. While respecting the authors' effort, there are some comments we would like to raise related to this review. The main aim of the review [1] was to focus on the current evidence on the associations between microbiota profiles and individual phenotypes and on the effect of bariatric surgery on gut microbiota.

First, the authors did not assess the methodological quality of the included studies. Systematic reviews have become increasingly important in healthcare and are considered the “gold standard” form of evidence for assessing the effectiveness of therapeutic interventions. One of the key steps in a systematic review is the assessment of methodological quality for eligible studies. A clinical study with inappropriate study design may result in multiple biases, such as selection bias, performance bias, attrition bias, detection bias, and reporting bias [2]. Generally, the reliability of the results of a systematic review depends on the extent to which potential sources of bias of included studies have been avoided [2]. The aim of bias assessment in a systematic review is (1) to identify the strengths and limitations of the eligible studies, (2) to investigate and explain potential heterogeneity in results across different studies, and (3) to grade the strength of evidence for a given question [3]. In addition, the concluded level of evidence in systematic reviews is an important source for both future research and clinical recommendations. Numerous tools can be used for assessing the methodological quality of clinical trials [4, 5]. For example, the Cochrane risk of bias tool was designed by the Cochrane Collaboration for use in systematic reviews of randomized controlled trials [2]. The Newcastle-Ottawa Scale is comprehensively used for nonrandomized studies, specifically cohort and case-control studies [6].

Second, the authors did not report the publication bias and heterogeneity. We suggest that publication bias should be assessed by statistical tests (e.g., Egger's linear regression test or Begg's rank correlation test), as well as the I^2^ statistic to quantify heterogeneity.

Third, the authors did not follow the Preferred Reporting Items for Systematic Review and Meta-Analysis (PRISMA) [7]; also, the inclusion criteria are ambiguous. The authors do not sufficiently follow the PICO format (participants, intervention, comparison, and outcomes). The authors state the inclusion criteria as “We assessed observational human studies or clinical trials that evaluated the gut microbiota of individuals who suffered from obesity. Obesity was defined by body mass index (BMI). We also selected observational studies of extreme weight loss interventions, such as bariatric surgery, but did not include dietary interventions, because there is a lack of homogeneity, and many reviews have already focused on this theme.” However, the review did not clearly report the other components of inclusion criteria like comparison and outcome measures. Running a systematic review without full knowledge about the inclusion criteria can lead to problems with assessing the validity, applicability, and comprehensiveness of the systematic review [7].

Finally, the search string was reported in the review ““Obesity” and “Microbiota”.” The authors did not include a detailed search statement in the supplemental materials; so, readers have to assume that the search in “Methods” represents the true extent of the search strategy. We suggest that the authors provide us with a complete search strategy to strengthen the credibility of the review.
